# Severe pneumonia with co-infection of H5N1 and SARS-CoV-2: a case report

**DOI:** 10.1186/s12879-023-08901-w

**Published:** 2024-01-02

**Authors:** Ke Jin, Zixing Dai, Ping Shi, Yuwen Li, Chuanlong Zhu

**Affiliations:** 1 Department of Infectious Disease, The First Affiliated Hospital of Nanjing Medical University, Nanjing, 210029 China; 2 Department of Pediatrics, The First Affiliated Hospital of Nanjing Medical University, Nanjing, 210029 China

**Keywords:** Pneumonia, H5N1, SARS-CoV-2, Co-infection, Antiviral agents, COVID-19

## Abstract

**Background:**

The H5N1 influenza virus is a cause of severe pneumonia. Co-infection of influenza virus and severe acute respiratory syndrome coronavirus 2 (SARS-CoV-2) may lead to poor prognosis of patients during the COVID-19 epidemic. However, reports on patients co-infected with avian influenza virus and SARS-CoV-2 are scarce.

**Case presentation:**

A 52-year-old woman presented with a fever, which has persisted for the past eight days, along with worsening shortness of breath and decreased blood pressure. Computed tomography (CT) revealed an air bronchogram, lung consolidation, and bilateral pleural effusion. The subsequent polymerase chain reaction (PCR) of the bronchoalveolar lavage fluid (BALF) revealed positivity for H5N1 and severe acute respiratory syndrome coronavirus 2 (SARS-CoV-2).

**Conclusion:**

The H5N1 influenza virus is a cause of severe pneumonia. The clinical presentation of the patient had a predomination of H5N1 influenza rather than COVID-19. A PCR analysis for the identification of the virus is necessary to reveal the pathogen causing the severe pneumonia. The patient exhibited an excellent prognosis upon the use of the appropriate antiviral medicine.

**Supplementary Information:**

The online version contains supplementary material available at 10.1186/s12879-023-08901-w.

## Background

Avian influenza virus (AIV) is a significant threat to public health as it is associated with a high mortality rate, and several novel variants of AIV also keep emerging [1]. The H5N1 virus is a subtype of AIVs that has been circulating among wild birds for the past few years. The transmission mainly occurs from animals to humans, animals to animals, and the environment to humans [2]. In humans, infection with H5N1 commonly develops into respiratory stress and pneumonia [3]. The first case of the transmission of this virus from poultry to humans was reported in 1997 in Hong Kong. Since then, several intermittent outbreaks have been reported in the human population throughout the world.

SARS-CoV-2 is another virus that has become a constant threat to global health since 2019, and it also leads to varying degrees of pneumonia [4]. Co-infection with this virus, particularly with the pathogens responsible for pneumonia, has been attracting great attention since the beginning of the pandemic of 2019. According to studies, co-infection of SARS-CoV-2 with different influenza viruses leads to a higher fatality rate compared to infection with the COVID-19 virus alone [5]. However, an extensive review of the literature revealed no case reports of such co-infection in which the category of the influenza virus was identified. In this context, the present report discusses a case of severe pneumonia caused by a co-infection of H5N1 and SARS-CoV-2 in a patient in China, the nation currently witnessing another surge in the cases of COVID-19.

## Case presentation

A 52-year-old woman who has been living as a countryside resident of the Anhui province in China since her retirement developed a fever on 1 February 2023. The patient visited a local hospital, where she was prescribed antibacterial treatment using piperacillin/tazobactam and levofloxacin. However, two days later, her symptoms worsened, and she experienced shortness of breath and decreased blood pressure. The patient was immediately transferred to the emergency department of the First Affiliated Hospital of Nanjing Medical University. According to records, the patient had no history of smoking, hypertension, or diabetes upon admission.

**Fig. 1:**
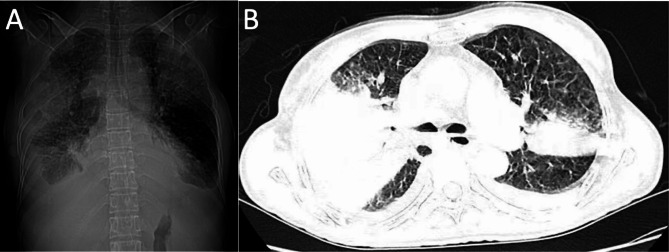
CT images prior to and after the treatment of the patient with the co-infection of H5N1 and COVID-19. The radiological examination of the patient after admission revealed an air bronchogram, lung consolidation, and bilateral pleural effusion (**A** and **B**)

The examinations performed at admission revealed a body temperature of 39 °C, worsening shortness of breath, a blood pressure of 104/67 mmHg, a heart rate of 103 bpm, and PaO_2_/FiO_2_ of 86 mmHg. The laboratory examination results revealed elevated C-reactive protein levels, procalcitonin lymphocyte count, and D-dimer levels (Table 1). The throat swaps for COVID-19 were negative. The coronal CT of the lung depicted multiple patchy shadows (Fig. 1A). In the axial imaging, air bronchogram was predominant in the upper lobe and the right middle lobe, along with lung consolidation and bilateral pleural effusion (Fig. 1B). Without delay, the patient was placed on a non-invasive ventilator and methylprednisolone (40 mg once daily) for the management of the severe acute respiratory distress syndrome. Subsequently, sputum samples were collected from the patient and analyzed. The culture, bacterial PCR, and fungal PCR of these sputum samples were negative. The immunological tests for tuberculosis and common respiratory pathogens were also negative. Thereafter, on the night of 7 February 2023, the patient appeared irritable and was transferred to the intensive care unit (ICU).

**Table 1:** Laboratory result on admission

Parameter	Result	Reference range
White blood cell	3.09 × 10^9	3.5–9.5 × 10^9/L
Lymphocyte count	0.53 × 10^9	1.10–3.20 × 10^9/L
Neutrophil count	6.95 × 10^9	1.80–6.30 × 10^9/L
D-dimer level	2.14	0-0.5 mg/L
C-reactive protein	88.7	0-10 mg/L
Procalcitonin	4.82	0-0.5ng/ml

In the ICU, the patient was placed on intratracheal intubation and mechanical ventilation. BALF samples were retrieved and subjected to metagenome next-generation sequencing (mNGS). The mNGS results were obtained three days after admission to the ICU, and the results were positive for H5N1 (Supplementary material). Therefore, a PCR analysis of the sputum and BALF samples from the patient was conducted for further identification of the virus, which confirmed the presence of the H5N1 influenza virus and presented a positive result for SARS-CoV-2. Accordingly, co-infection of H5N1 and SARS-CoV-2 was identified as the etiology of severe pneumonia in this patient. The patient was then administered Peramivir (0.6 g once daily) and nirmatrelvir-ritonavir (300 mg/100 mg, every 12 h) for the next 5 days.

**Fig. 2:**
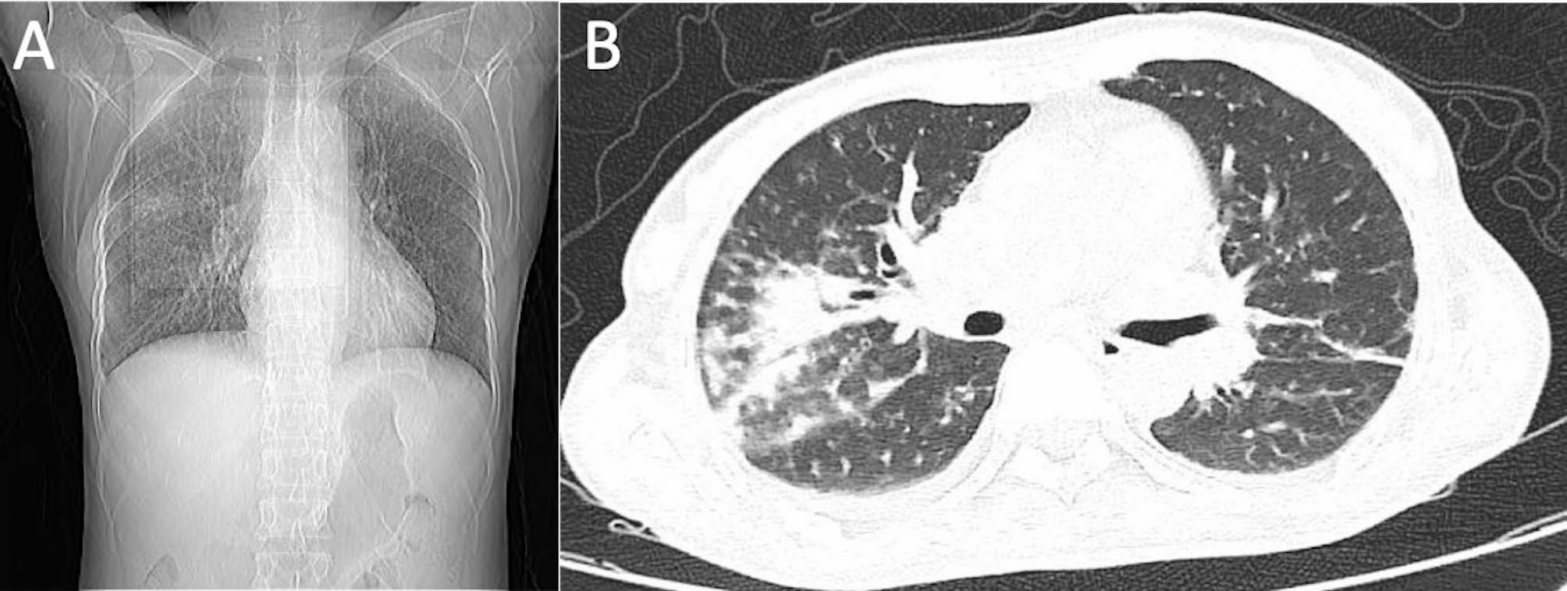
Ten days after treatment with the antivirals, the lung inflammation was relieved in the patient (**A** and **B**)

After treatment with the above antivirals, the inflammation indices and the temperature of the patient improved. Thirteen days after admission to the ICU, the pulmonary inflammation had reduced, as evidenced by the CT images (Fig. 2A and B). Accordingly, mechanical ventilation was withheld, and the corticosteroid was discontinued. Twenty-seven days after admission to the ICU, the patient tested negative for both H5N1 and SARS-CoV-2 and was, therefore, discharged from the hospital.

## Discussion and conclusions

The PubMed database was searched using the terms “H5N1”, “SARS-CoV-2”, and “co-infection”, and this search revealed no articles on patients having a co-infection of H5N1 and SARS-CoV-2. According to previous reports, under proper supervision, the incidence rate of avian H5N1 influenza in humans has been decreasing in the past few years [6, 7]. However, the mortality rate due to severe acute respiratory distress induced by the H5N1 virus continues to be high [3]. In China, the implementation of “zero COVID” strategies was abandoned on 7 December 2022, after which a surge was recorded in the cases of infection with the Omicron variant of the SARS-CoV-2 [8]. Co-infection of the COVID-19 virus, particularly with the other pathogens responsible for pneumonia, has been attracting great attention since the beginning of the COVID-19 pandemic. According to reports, co-infection of SARS-CoV-2 with different influenza viruses leads to a higher fatality rate compared to infection with the COVID-19 virus alone [5]. Numerous cases of infection with both SARS-CoV-2 and influenza A virus were reported during the COVID-19 epidemic, while cases of co-infection with SARS-CoV-2 and H5N1 were scarce [9]. In the case discussed in the present study, no other member of the patient’s family was infected with H5N1, except for the patient, who had been exposed to sick poultry. Therefore, it was understood that the spread of the virus was limited to animal-to-human transmission only. Accordingly, it is recommended to ensure the protection of the upper respiratory tract of humans against droplets containing AIVs, particularly during contact with infected chickens and birds.

The CT images of the patient revealed interstitial infiltrates, lung consolidation, diffuse ground-glass opacities, and air bronchogram, all of which are symptoms observed during a common viral infection [10]. The typical features of COVID-19 in CT images include bilateral multi-lobar ground-glass opacities, which were not detected in the CT images of the discussed patient. The WHO has defined two types of SARS-CoV-2 variants: the variants of concern (VOCs) and the variants of interest (VOIs). Several VOCs have caused multiple waves of epidemics, including Alpha (B.1.1.7), Beta (B.1.351), Gamma (P.1), Delta (B.1.617.2), and Omicron (B.1.1.529) [11]. The Omicron variant was first detected in China in December 2021. The prevalence of the Omicron variant in patients presented with a greater frequency of bronchial wall thickening and less-typical CT patterns [12]. However, the lesions occurring simultaneously in the central and peripheral regions of the lung and pleural effusion were uncommon in the cases of COVID-19. The clinical presentations were the same as those observed in the cases of isolated H5N1 infection and atypical for the pneumonia of COVID-19. Higher viral loads were evident in the BALF compared to the nasopharyngeal samples. Therefore, a single examination for the influenza virus, such as PCR for throat swabs, usually exhibited a relatively low sensitivity. [3]. In the event of viral pneumonia, timely antiviral treatment is key to decreasing mortality [9]. Therefore, for the case discussed in the present report, the mNGS of the BALF samples and the PCR test were performed at the earliest to verify the diagnosis, and this played a vital role in elucidating the etiology of viral pneumonia [13]. After the diagnosis, considering that the infection was caused by two viruses, the corresponding two categories of antiviral medicine were prescribed. While previous studies have demonstrated that co-infection with influenza and COVID-19 leads to a poor prognosis, the administration of both antiviral and anti-inflammation treatment in the present case could relieve lung inflammation, leading to an excellent prognosis.

In conclusion, it is important to state that co-infection of H5N1 and SARS-CoV-2 in patients may not lead to a terrible prognosis if timely treatment is administered. Indeed, influenza A virus infection can elevate ACE2 expression to promote the infectivity of SARS-CoV-2. [14] However, despite the absence of any severe or adverse events in the disease course of the present case that had a co-infection of H5N1 and SARS-CoV-2, it is recommended to ensure further precise treatment by verifying the pathogen responsible for causing severe pneumonia through various examinations.

### Electronic supplementary material

Below is the link to the electronic supplementary material.

Supplementary Material 1

## Data Availability

The datasets used and/or analyzed during the current study are available from the corresponding author on reasonable request.

## Abbreviations

SARS: CoV-2 Severe acute respiratory syndrome coronavirus 2
CT: Computed tomography
PCR: Polymerase chain reaction
BALF: Bronchoalveolar lavage fluid
AIV: Avian influenza virus
ICU: Intensive care unit
mNGS: Metagenome next-generation sequencing
