# Supplementary material for: Severe pneumonia with co-infection of H5N1 and SARS-CoV-2: a case report
Source: BMC Infect Dis. 2024 Jan 2;24:31. doi: 10.1186/s12879-023-08901-w (PMC10763204; doi:10.1186/s12879-023-08901-w)
Supplement: Supplementary file 1 — Supplementary Material 1 [file 12879_2023_8901_MOESM1_ESM.docx]

**Supplemental material**

Samples of bronchoalveolar lavage fluid were used for metagenomic next-generation sequencing which presented eight sequences consistent with H5N1 virus. These sequences are as followed.

>NODE_3_length_2357_cov_48136.804217 GTCGTTTTTAAATAATTCGACACTAATTGATGGCCATCCGAATTCTTTTGGTCGCTGTCT GGCTGTCAGTAAGTATGCTGGAGTCCCGTTTCCGTTTCATTACCAACACCACGTCTCCTT GCCCTATCAACACATTAGCCTTTTCCCCTTTCGCAAGACTGCTCAATTCGTTGATGCTTA GTGCTGGTCCGTATCTTTTGTCCTCTTTGCCCAGAATTAGGAATCCTCTCAATACTGCAG ACTCCACTCCTGCTGTTCCCTCATCTGGATCTTCTGCCAAGGCACCTGCATCTTTTCCAA GAACTGTGAGTCTCTTGGTTGCCTTGTTATAGTTGAACACAGGGGAGTTGCCTCTCACAA GTATCCTCATTCCTGAACCTCTAACGTTCACAGTCAGAGAGGAAAACTGCATTCTACTTT GTTCCGGTGGGGCTGCTGCAAATGGTAGGAGCTTTATTATTTGTACAGTGTCGAATGTTC CCAGCACATCACGCATCTGCTGGAACAATGTCCTTACAAATCCACTATACTGGCCTCTGG CAGCCTTGGGCACCAAGGATTGAAAGGGCTCAAATTCCATTTTATTGTACAACATCGTAG GGTCTTGGGACCATTGAATCTTCACAGTTTCCCAATTTCTAATGATCCACTGATATGTGT TGACTAGCACGGATTCCGGACCATTGATTTCCCACATCATAGACGATGAATATGTTATCG TCAGTTTCTCTGTTCCCTGTGTTTCACTAACTTCCTCAGGAGACAGGAGCACGTTCCCTC TCTGATCTCGGACTCTCAAGAAGCGATCAATGCTCACGACCACTCTCTCAGTACTGGAAT ATTCATCCACTCCCATTTTGCTTACTCTCACTCCTCTTAGTGACATCTCTGTGCTGGGGG TCATGTCAGGCATTATTCCAATCATCCCCATTACATTGTCAATTGGTTCAATTCCCCAGT TTTGAAACAGCACCTTTGCATCCTTTTGGAAATGCCTCAGGAGTTGATGCATAGGATTTA GCCGCTGGTTCGCTCTGTTAACAAAATTCAGATCACCTCGTACTGCTTTTATCATGCAGT CCTCTTGTGAGAAAACCATTGCCACTATGATTGCTTCGGCGATTGACTGCTCGTCTCTCC CACTCACTATTAATTGGATCAGCCTTCTGGTTGCTTTTCTCAAAATGGCTGTAGCTCTTC GCCCGACCATTGTGAATTCCTCATATCCTTCGTGCACTCTTATTTTCAGTGTTTGGAGGT TGCCTGTGAGCACTTCTTCTTCTCTTTTGTCAGATGACCCACTTGTCCTTTTGAAAGTGA AACCTCCAAAGCTGAAGGATGAACTGATTCTTAAGCCCATTGCTGCTTTGCATATATCCA CAGCTTGCTCTTCTGTTGGATTCTGTCTAAGAATGTCCACCATCCTTGCTCCGCCAATTT GTGTACTGTGGCACATTTCCAAGAGCGAAGCCAATGGGTCTGCTGATACTGTTGCTCTCC TAACGATATTTCTAGCAGCAATGATCAAACTCTGATCAAGATCATCATTTCTCACCTCCC CTCCCGGTGTGTACATTTGTTCCCAGCAGGTCCCTTGAGTCAAATGCAATACCTCGATAT ATACGCTGCTTGTTCCTCCAGCTACTGGTAAAAATCTGGTTTTGCGAACCAGTTCCCTCT CCAACATGTATGCCACCATCAAAGGAGCAATCTTACAGTCCTGAAGTTCCTCCTTCTTTT CCTTTGTTATTGTTAATTGTGATTCTGATGTCAATATCCTGGCTCCAACTTCATTTGGGA AAACGACCTCCATGATGACATCCTGTGCTTCTTTGGCACTGAGATCTGCATGGCCCGGGT TTATGTCAACCCTGCGGCGTATCTTAACCTGATTTCGAAAGTGAACAGGGCCAAAGGTTC CATGTTTCAACCTTTCTACCTTTTCAAAGTAGGTTTTGTAGACCTTTGGATAGTGGACTG TACTTGTCGTTGGCCCATTTCTATTCCACCATGTTACAGCCAGAGGTGACACCATCACTC TGTCTGATCCAGCGTCATTTGTTTTGCTCCAGAGAGTCTGACCTTGCTCATTCCTTTCAG GGATCATCTCCATTATTCTTTTGTCTGCTGTAATAGGATATTTCATTGCCATCATCCATT TCATCCTGAGGGCAGGGTTCTTCTCCTGTCTTCCTGATGTGTACTTCTTTATTATGGCCA TATGGTCCACAGTGGTTTTTGTCAGTATCTCGCGAGTGCGAGACTGTGACATTAAATCTC TTAGTTCTTTCATTCTCTCCATATTGAATATATTTGACCTGCTTTCGCTGGATCAGACAG AGTGATGGTGTCACCTC

>NODE_4_length_2322_cov_36775.396680 TCATGAAGGACAAGCTAAATTCACTATTTTTGCCGTCTGAGCTCTTCAATGGTGGAACAG ATCTTCATGATCTCAACAAACTCTTCCTTCTTGATCCTTCCAGATTCGAAATCAATGCGT GCATCGATTCGGGCCCTAGACACCATGGCCTCCACCATGCTGGAAATTCCAACTGGTCTC CTGTATGAACTACTGGGGAAGAATTTCTCGAATAGACTGCAGCATTTCTGGTACATCTGT TCATCCTCAAGAATTCCCCTTTGACTGGTATTGAGAATGGAACGATTCCTTTTAGGAATC CATGAGTGTGTAGTCGCAACGGCGTCATATTCCATGCTTTTGGCTGGACCGTGGGCTGGC ATCACCACAGCATTGTTTACGGACTCAATCTCTTTATGGCTGACAAATGGGTTCAGAGGA TTACACAACCTACCCTGGTAATCTTCGTCCATCAGCTCCCACTTCAGGCAGACCTCTGGG ATATGGAGATTCCGAATGTTGTATAGATTTGGTCCTCCATCTGACACCAACAGTCCTGCC TTAGAACGGGTCTGTTCCCACAGCTTCTTCAGCTCGAATGATCTCCTCGTTTGAATTTGT GTGTCACCTCTGTGGCATCGGTACGTGTACCTGTAATCTTTGATGAATAGTTGAAGGGCC ATTTGAGCTGTTGCTGGTCCAAGGTCATTGTTAATCATGTTGTTCTTTATCACTGTGACG CCAATGCTCATGTCAGCTGATTCATTGATCCCAGAAACTCCAAAGCTGGGCAGCTCCATA CTGAAGTTAGCCACAAATCCATATCGATAGAAAAAGCTTGTGAACTCAAATGTTCCAGTC CGGTTTATGTAAGACTTCTTTTTGCTCATATTGATCCCGACCAGTTTGCAGGTCCTATAG AACCTATCAACTCCTGCTTGTATTCCCTCATGATTCGGTGCATTTACTATGAGGGCGAAA TCATCAGAGGATTGGAGTCCATCCCACCAGTATGTGGTTTTGGTGTACTTCTTTTGCCCA AGATTTAGGATTGAAACTCCTAATACTGTACTCAGCATATTGAACATGCCCATCATCATT CCAGGACTCAATGAGGCAGTGCCATCTATTAGTAGAGGCCTTATTTTTTCGATTTTCTTT CTTGTTGATTCATTGAAGTATCTTAAGTCAATGTTTGCAAGCATTTCTGCAGGTATTTGT GTCCGTAACTTCATGCTCTTACTTTCAAACATGTACCCTTTTCCCAATCTCGCCATTTTG TTCGAGAACATTATAGGGGCAATACTCAAGACATTTCTAAACCATTCGGGTTGGTTTCTT GTAATATATGTTATCATTGCCAGAAACATCCGAGGATTTTGATTCTCATTCCATTTGGTG TTATCTCCAGTAATTGTGAAGGAGAGCTCTGTATCTTGTGAGTTAGTCATCATTTTTCTC ACGACATTTGCCAATTTAGCCTTCTTCTCATTCCCTCCAACGGGGAGTCCAGATTGCTCA AGTTTTTCACAGATGCTCCTTGCCAGTGTTTCGACAAAGTACACAAATCCTCTAATCTGC ATCCCTGGTGTTGCAATCGCCCGTCTCTTCAATTTGCCTCTTTCTGCATCTTTTGTCATT GTGTTCAGTGTCAGTGCTCTTATTAAGTAGCTCCTCTTGTTTAGCCTTTGTTTCTTCTTT CCTATTGTTCTTTGTGTGACCATTTTCTTGGTCATGTTGTCCCTTACTCTTCTCTTTCTC TGGAAATGTGTTGTTATTTCCATTTCTTCTTTATCCATTGAGTCCATCACATCCTTGAGG AAATCTATTAGTCTCCCCGATTCATTGGCTGTTAGACCGTTTGATCTGAAGACCTCTATA GTGTTGGCCAGGGCAGTTGCAGCTGGTTGGTTTCGATTCAGTGTCCAGTCATACGTCTGG CGACCTTGAGTTAGTTTGTCCACTCTTGTTTGCTGAACGACTTCCATCGTTTCAAGACAA GAGTTTTCAAAGATCCCTGGGTGGGACTCTTCAAGGAAAGCCATTGCTTCCAACACGCAA

TCTGTTTGTGCATATCCGCTCGGTTCGTTGTCCTCAGGTAGTGGCCCATCAATTGGGTTG AGTTGAGGTGCTCCGGTTTCTGTGTTTGTTGTCCATTTTCCCTTCTCTGAGTATTGATGT GTTCTGTTGACTGTGTCCATTGTGTATCCTGTCCCTGTTCCATGGCTGTATGGAGGATCT CCAGTATAAGGGAATGTGGTACTTATGGCATCTTGCGCTGGCACTTTTAAGAAAAGTAAA GTCGGATTGACATCCATTCAAATGGTTTGCCTGCTTTCGCTT

>NODE_5_length_2206_cov_58659.198803 TAGACCTCTACTGGCCATTTCTTGTCTTATAGCGAAAGCAGGTACTGATCCAAAATGGAA GACTTTGTGCGACAATGCTTCAATCCAATGATTGTCGAGCTTGCGGAAAAAGCAATGAAA GAATATGGGGAAGATCCGAAAATCGAAACAAACAAATTTGCCGCAATATGCACCCACTTA GAAGTCTGTTTCATGTATTCGGATTTCCATTTTATTGATGAACGAGGCGAATCAATGATT ATAGAATCTGGCGATCCAAATGCATTATTGAAACACCGATTTGAGATAATCGAAGGGAGA GACCGAGCAATGGCCTGGACAGTGGTGAATAGTATCTGCAACACCACAGGAGTCGAAAAG CCCAAATTCCTCCCTGATTTGTATGACTACAAAGAGAACCGATTCATTGAGATTGGAGTA ACGCGAAGGGAAGTTCACATATACTATTTAGAAAAAGCCAACAAGATAAAATCAGAGAAA ACACACATTCACATATTCTCATTCACTGGAGAGGAAATGGCCACCAAGGCGGAATACACC CTTGATGAAGAGAGCAGAGCAAGAATAAAAACCAGACTGTTCACTATAAGACAAGAAATG GCCAGTAGAGGTCTATGGGATTCCTTTCGTCAGTCCGAGAGAGGCGAAGAGACAATTGAA GAAAGATTTGAAATCACAGGAACCATGCGCAGGCTTGCCGACCAAAGCCTCCCACCGAAC TTCTCCAGCCTTGAAAACTTTAGAGCCTATGTGGATGGATTCGAACCGAACGGCTGCATT GAGGGCAAGCTTTCTCAAATGTCAAAAGAAGTGAACGCCAGAATTGAGCCATTTCTGAAG ACAACACCACGCCCTCTCAGATTACCTGATGGGCCTCCTTGTTCTCAGCGGTCGAAGTTC TTGCTGATGGATGCCCTTAAGTTGAGCATCGAAGACCCTAGCCATGAGGGGGAGGGCATA CCGCTGTATGATGCAATCAAATGCATGAAGACATTTTTTGGCTGGAAAGAGCCCAACCTC GTAAAGCCGCATGAGAAAGGCATAAACCCTAATTACCTCCTGGCTTGGAAGCAGGTGCTA GCAGAACTTCAAGATATTGAGAATGAGGAGAAATTTCCAAAAACAAAGAACATGAAGAAA ACAAGCCAATTGAAGTGGGCACTTGGTGAGAACATGGCACCAGAAAAAGTGGACTTTGAG GACTGCAAAGATGTTAGCGATCTAAAACAGTATGACAGTGACGAACCAGAGCCTAGATCA CTAGCAAGCTGGATTCAGAGCGAATTCAACAAGGCATGCGAATTGACAGATTCAAGTTGG ATTGAACTTGATGAGATAGGAGAAGATGTTGCTCCAATCGAACACATTGCGAGTATGAGG AGGAACTATTTCACAGCGGAGGTATCCCATTGCAGGGCCACTGAATACATAATGAAGGGA GTATACATAAACACAGCTCTATTGAATGCATCCTGTGCAGCCATGGATGACTTCCAACTG ATTCCAATGATAAGCAAATGCAGAACCAAAGAAGGAAGACGGAAGACAAATCTGTATGGA TTCATTATAAAAGGAAGATCCCATTTGAGGAATGACACCGATGTGGTAAACTTTGTGAGC ATGGAATTCTCTCTAACTGACCCGAGGCTAGAGCCACACAAATGGGAAAAGTACTGTGTT CTTGAGATAGGAGACATGCTCCTACGGACTGCAATAGGCCAAGTGTCGAGGCCCATGTTC CTGTATGTGAGAACCAATGGAACTTCCAAGATCAAAATGAAATGGGGCATGGAGATGAGA CGGTGCCTTCTTCAGTCCCTTCAACAAATTGAGAGCATGATTGAGGCCGAATCTTCTGTC AAAGAGAAGGACATGACCAAGGAATTCTTTGAAAATAAATCAGAAACATGGCCAATTGGA GAATCACCCAAAGGGGTGGAGGAAGGCTCTATTGGGAAAGTATGCAGAACATTGCTAGCA AAGTCTGTGTTCAACAGCCTATATGCCTCTCCACAGCTCGAGGGATTTTCAGCTGAATCA AGAAAATTACTTCTCATTGTTCAGGCACTTAGGGACAACCTGGAACCTGGAACCTTCGAT CTTGGGGGGCTATATGAAGCAATTGAGGAGTGCCTGATTAACGATCCCTGGGTTTTGCTT AATGCGTCTTGGTTCAACTCCTTCCTCACACATGCACTGAAATAGT

>NODE_7_length_1810_cov_9054.004502 CATTTAGATTTGTGAGCTCAGATTGTAGTAGAAACAAGGGTGTTTTTAACTACAATCTGA GCTCACAAATCTAAATGCAAATTCTGCACTGTAACGACCCATTGGAGCACATCCATAAAG ATAGACCAGCCATCATGATTGCCAGTGCTAGGGAACTCGCCGCTGTTGAATAAATTGACA GTATCTGGTAAGTTCCTATTGATTCTAATTTCACTCCGCTTATTTCTTCTCTTTTTAATC TTGCTTCTTCTGAATACTGAGGGTAGTAATACGTCCCATTTCTCACACTTTCCATACATT CATTATCGCATTTGTGATAGAATTCGAAACAGCCATTACCCAGCTCCTTTGCATTATCCC TAAGCTGTAGTCTGACTTTGTCGTAAAGGTTCTTGACATTTGAATCATGGAAATCTAGAG TCCTCTCGTTTTCCATGAGAACTAGAAGTTCAGCATTATAGGTCCAGACATCTAGGAATC CGTCTTCCATTTTCTTGTTCAAATTCTCTATCCTCCTTTCTAAGTTATTAAATTCCCTTC CAACTGCCTCAAATTGAGTGTTCATTTTGTCAATGATTGAGTTGACCTTATTGGTAACTC CATCTATTGCCTTTTGGGTGGATTCTTTGTCTGCAGCGTACCCACTTCCCTGCTCATTGC TATGATGGTACCCGTACCAACCATCAACCATCCCCTGCCATCCTCCCTCTATAAACCCTG CTATCGCCCCAAACAGGCCTCTTTTTCTTCTCTTTTCTCTTGGAGGACTATTTCTGAGCC CAGTCGCAAGGACCAACTTGTTTGACTTCACGTATTTGGGGCATTCCCCAATGGTGAGAG GATGTATGTTGTGGAATGGCATACTAGAATTTATCGCTCCTACTGGGGTTTGACATTTGG TGTTGCAGTGGCCATATTCCACTCCACTTTTCATAATTGTTGAATCCCCTTTCTTGACAA TTTTGTATGCATATTCTGGAGCAATGAAATTTCCATTACTCTCGAAATGGATTGCATCAT CTGGTTTTAAAATTGTCCAGAAGAAGTCCATTCTTCCACGTTGCCCATTTACTTGGGATC TAGTAGCTATTTTTGGTACCAATCTCTGATTTAAAGTTGATGTTCCAACTGAAATGTAGG TGGTTGGGTTTTTATAGAGATTTGTCTGCTCTTCTGCATTGTTAGAATGATGAATCCCCC ACAGTATCAAGAGATCTTCCCGATTGGTATTATTGTAGCTTATCTTTATTGTTGGGTATG CATCGTCCTTTTTGATAAGCCACACCACATTTCTGAAAAAGGAGGGTGCTCCCTGGTATG GACAAGCTGCGCTCACCCCTAGTGATGTTTCATGATTTGGCCAGGAACTCTTGGGGATGA TCAGAATCTTCTCAAAATGATTTATTCTGCTCAACAGGTGTTTCAGTTCTTCATAGTCAT TGAGGCTCCCTGGGTAACAGAGGTCATTAGCTGGATTATCCCGCTCCACTATGTAGGACC ATTCCGGCACTCTGATGAATTCGTCGCACATTGGGTTTCCGAGGAGCCATCCAGCTACAC TACAGTCCTTTAAAATCAGAGGCTTCACCCCATTTAGATCACAGAGCTTCCCGTTGTGTG TTTTCTCCAGTATGTCTTGGGCATGTGTAACAGTGACGTTCTTTTCCATTATCGTGTCAA CCTGCTCTGTCGAATTGTTTGCGTGGTAACCAATGCAAATCTGATCACTTTTGACAAGGT TAACTATTGCAAGAAGAAGTACTATGTTCTCCATTTTGACAGAGTGAACCCCTGCTTTTG CGGCAATGAA

>NODE_10_length_1492_cov_137762.442084 TACTCCTCTGCATTGTCTCCGAAGAAATAAGATCCTTCATTACTCATGTCAAAGGAAGGC ACGATCGGGTTCGTTGCCTTTTCGTCCGAAAGCTCGAAGACTCCCCGCCCCTGGAAAGAC ACATCTTCTGGTCTGGCACTTTCCATCATTCTTATGATTTCAGTCCTCATGTCGGATGTT CTGCCCTCAGTATTCCCTGTGAACGCCGCCATAATGGTCGCTCTTTCAAAGGGAAGATTT CTCTGTACCGAAAAAGTGGGCTGTACACTGATCTGTCCTGCCGATGCTCTCTGTTGGTTG GTGTTTCCTCCACTCCTGGTTCTTATAGCCCAATATCTGCTTCTCAGCTCAAGAGTGCTG GAGTCCATTGTTTCCATGTTCTCATTTGAAGCAATTTGAACCCCTCTTGTGGATAGTCGT CCTCTTGGAACTACTCTTGTTCCTCTGATGAAACTTGAGACTCTCAGATCCTCGAATGCT GCGGAATGACATGCCATCCATACCAATTGACTTTTGTGTGCTGGATTCTCATTTGGTCTA ATGAGGCTGAAGACCTGGCTGTTTTGGAGCAGACGGAAAGGATCTATCCCAACTAGAGAG TACCCTTCTCTCTCAAAGTCGTATCCACTGGCCACAGCGAGTCCGTACACACAAGCAGGC AAGCAGGACTTATGAGCCACTGATCCTCTCAGGATGAGTGCAGACCGTGCCAGAAAGATG AGATCTTCAATTTCAGCATTGCCAGGATTCCTGCTTTCACGCACCTGGTCCATCATTGCT CTTTGCGCTGCTGTTTGGAATTTCCCTTTGAGGATGTTGCACATTCTCTCATATGCAATC CTTGTCCTTCGTCCGTTCTCGCCTCTCCAGAAGTTCCGATCATTAATTCCTCGCTTTATC ATCCGAATTAGTTCCATCACCATCGTCCCGACTCCCTTTACTGCTGCACCAGCAGCTCCA GACCTCCTTGGGAGAGTTGATCCTTGCATAAGAGAGCACATCCTGGGGTCCATCCCAGTA CGCACGAGAGCTCTTGTCCTCTGGTATGTGGCATCATTTAAATTAGAATGCCAGATCATC AAGTGAGTGAGACCAGCAGTTGCGTCTTCTCCATTGTTCGCTTGACGCCAGATTCTCCTG ATCTCCTCTTTGTCATACAGGATCAGTTCTCTCACCCATTTCCCATCTCTCCTTCGATAA ATTGGACCTCCAGTTTTCTTCGGGTCCTTCCCCGCACTGGGGTGTTCTTCCAGATATTTG TTCCTCCTTTCATCAAATGCAGAGAGAACCATTCTCTCTATTGTTATGCTGTTCTGGATC AGCCTTCCTTCGTGGTCGCTGAGTTTGAGCTCAGTGCACATCTGTATGTAGAACCTCCCA ATTCCACCAACCATTCGTCCAACAGATGCTCTGATCTCAGTGGCATTCTGGCGCTCTCCA CCAGTCTCCATCTGTTCATAGGATCGTTTGGTGCCTTGAGACGCCATGATGT

>NODE_14_length_1346_cov_25271.849200 AAACTACTTGTCAATGGTGAATGGCAACTCAGCACCGTCTGGCCAAGACCAACTCACAGT GTCACTATTTACACCACAAAAGGATATGCTGCTCCCGCTAGTCCAAATTGTGTTCTCTTT GGGCCTCCCTCTAATCAGCTCAACCCAGAAACAAGGCCTCATGCAATCTAATCCTGTCAG TTCTGGGTGCTGGACAAAAGTCCCACTATATCCTGACCAGTCAGTTATTTCTATGATGTC TTGCTTCATTGAGAAACTACTGTCCGTCTCAGTCCACCCATTCGGATCCCAAATCATCTC AAAGCCGCTTCTGGAACTAGTGCTCTTTGTTCTTCCGATCCAAACCCCATTACCGTACTT AAATGAAAACCCTTTTACCCCATATGCCCCGTTAGAGGACATTGGACTGCAACTGCCTGT TCCATCATTGGGGCGGGGGTTGTCTCCGAAAACCCCACTGCATATATATCCTATTTGATA CTCCAGATTATGATTAAAAGATACCCACGGCCGGTTTGAGCCATGCCAATTGTCCCTGCA CACACACATAATCTCACCCGCATCAGGATAACAGGAGCATTCCTCGTAGTGGTAATTAGG GGCATTCAATTCAACTGATTTGACTACTTTCCCTTTCTCTATCTTGAAGATTTTATATGA GGCCTGCCCATTGCTTGGTCCATCAGTCATTACAGTGAAGCAAGAGCCATTTACACACGC ACATTCAGATTCTTGAGTTCTCAAAATGTTGTTTCTCCAACTCTTGATAGTATCTGTTAT TATGCCATTGTACTTCAATACAGCCACAGCTCCATTGTCTGGACCAGAAATACCGATTGT CAACCAACTGATGCCATCATGACAAGCACTTGCCGACCAAGCAACAGACTCAAATCTTGA ATTGTACGGGGAAGGAGCCTCACCCACGGGACAACTCATCAAAGTTCTATAGGGGCTTCT ATCCTTAACGGTCCCATTAGAATGTTTGTCATTCAGCAGAGCTCCCTGGGTCAGAAAAAA GGTTCTGCATTCTAAGTGGGAGCATGAGATGAACGGTTCTCTTATAACAAACACATCCCC CTTGGACCCAATTCTTATACCGTTGTCCTTACTGTATATAGCCCACCCACTAATAGGGCA AAGAGATGAATTGCCCGCTAATGTTACCGAAGTAACAGCCTGTTCAGCAAGAAAATTGGT ATTGTTGATGTTGACATACGTCTGATTTACCCAGGTGTTGTTCTCATAGGTAATGATGCT TTGATTGCATGTTTCAGGCTGGTGTTGATTCCCTGTTTGGATTGAATGGCTAACCCATAT TGAGATTATGTTCCCAATTTGCAGCA

>NODE_21_length_1027_cov_134752.088531 CGACCCAGCCATCTGCTCCATAGCAAAAGCAGGTAGATATTGAAAGATGAGTCTTCTAAC CGAGGTCGAAACGTACGTTCTCTCTATCGTCCCGTCGGGCCCCCTCAAAGCCGAGATCGC GCAGAGACTTGAAGATGTCTTTGCAGGGAAGAACACCGATCTTGAGGCTATCATGGAATG GCTAAAGACAAGACCAATCCTGTCACCTCTGACTAAGGGGATTTTGGGATTTGTGTTCAC GCTCACCGTGCCCAGTGAGCGAGGACTGCAGCGTAGACGCTTTGTCCAAAATGCTCTAAA TGGAAATGGAGACCCAAACAACATGGACAGGGCAGTCAAACTGTACAGGAAATTGAAGAG AGAGATAACATTCCATGGGGCTAAAGAAGTTTCACTCAGTTACTCAACCGGTGCACTTGC CAGTTGTATGGGTCTCATATACAACAGGATGGGGACGGTGACCGCAGAAGTGGCATTGGG CCTAGTATGTGCCACCTGTGAGCAGATTGCTGATTCACAGCATCGGTCTCACAGGCAGAT AGCCACCACCACCAACCCACTAATCAGACATGAAAACAGAATGGTGTTGGCCAGTACTAC AGCTAAGGCTATGGAGCAGATGGCTGGGTCGAGTGAGCAAGCAGCGGAAGCCATGGAGGT TGCTAGTCAGGCTAGGCAAATGGTGCAGGCGATGAGGACCATTGGAACTCATCCTAGCTC CAGTGCCGGTCTGAGAGATGATCTCCTTGAAAATTTGCAGGCCTACCAAAAACGGATGGG TGTGCAACTGCAGCGATTCAAGTGATCCTCTCGTTATTGCCGCAAGTATCATTGGGATCT TGCACTTGATATTGTGGATTCTTGATCGCCTTTTCTTCAAATGCGTTCATCGTCGCCTTA AATACGGTTTGAAAAGAGGGCCTTCTACGGAAGGAGTACCTGAGTCCATGAGGGAAGAGT ACCGGCAGGAACAGCAGAGTGCTGTGGATGTTGACGATGGTCATTTTGTCAACATAGAGC TGGAGTA

>NODE_25_length_920_cov_34186.925592 GCTTGACACAGTGTTGGAATCCATTATGTTTTTTATCATTAAATAAGCTGAAACGAGAAA GTTCTTATCTCTTGCTCCACTTCAAGCAATAGTTGTAAGGCTTGCATGAAAGTTATCTGT TCGAAACTGTTCTCTGTAATCTTCAATCTGTGCCGCACTTCTTCAATCAGCCATCTTATT TCTTCAAACTTCTGACTCAATTGCCCTCGCCATTTTCCGTTTCTGCTTTGGAGGGAGTGG AGGTCTCCCATCCTCATTACTGCTTCTCCAAGCGAATCTCTGTAGAGTTTCAGAGACTCG AACTGTGTTATCATTCCATTCAAGTCCTCCGATGAGGACCCCAATTGCATTTTTGACATC CTCATTAGTATGTCCTGGAAGAGAAGGTAATGGTGAGATTTCTCCCACAATTGCTCCTTC TTCTGTGAAAGCTCTAATTAGTATTAGGGTTTCCAGCCGGTCAAAAATCACACTGAAGTT TGCTTTCAATATGATGTTTTTATCCATTATTGCCTGGTCCATTCTGATGCAAAGGGAACC TGCCACTTTCTGTTTGGGCATGAGCATGAACCATTCCCTTGACATCTCCTCAAGAGTCAT GTCAGTTAGGTAGCGTGAGGCTGGCACGGGGGCAATGGTCATTTTAAGTGCCTCATCAGA TTCTTCTTCCAGAATCCGCTCCACTATCTGCTTTCCCGCACGGGTGGCTGTTTCGATGTC CAGACCAAGAGTGATGCCTCTTCCTCTCAGGGACTTCTGATCTCGGCGAAGCCGGTCAAG GAATGGGGCATCACCCAGTTCTTGGTCTGCAAATCGGTTGCGGACATGCCAAAGAAAACA GTCTACCTGAAAGCTTGACACAGTGTTGGAATCCATTATGTTTTTGTCACCCTGCTTTTG CTTTCAGGTAGACTGTTTTC
